# Potential new treatment for inferior vena cava injury using extracorporeal membrane oxygenation applying flow diversion effect

**DOI:** 10.1136/tsaco-2024-001618

**Published:** 2024-11-18

**Authors:** Takaaki Maruhashi, Keita Saku, Hideo Maruki, Marina Oi, Yasushi Asari

**Affiliations:** 1 Department of Emergency and Critical Care Medicine, Kitasato University School of Medicine, Sagamihara, Kanagawa, Japan; 2 Department of Cardiovascular Dynamics, National Cerebral and Cardiovascular Center, Suita, Osaka, Japan

**Keywords:** abdominal injuries, liver, Veins

## Abstract

**Background:**

Retrohepatic inferior vena cava (IVC) injuries remain among the most lethal and serious liver injuries. Gauze packing is currently the first choice for IVC injuries; however, laparotomy itself poses the risk of circulatory collapse. Thus, less invasive treatment strategies are needed.

**Methods:**

In this study, we conducted an animal experiment to replicate and validate successful treatments for an actual case of retrohepatic IVC injury that we had encountered.

**Results:**

A woman in her 80s presented to our hospital due to cardiac arrest caused by a pulmonary artery embolism. Venoarterial extracorporeal membrane oxygenation (ECMO) was introduced, and the patient was resuscitated. After resuscitation, contrast-enhanced CT revealed liver and retrohepatic IVC injuries, possibly caused by chest compressions. Liver injury was treated using transarterial embolization of the left hepatic artery. To treat the retrohepatic IVC injury, ECMO flow was increased to enhance the negative drainage pressure. The extravasation of the contrast medium had resolved in IVC angiography, and we opted for nonoperative management. The patient’s hemodynamic status gradually stabilized, and ECMO was withdrawn on day 6. We confirmed these findings in a dog model of retrohepatic IVC injury.

**Conclusions:**

Our findings from the patient and the animal model suggest that the flow diversion effect of ECMO can effectively manage active bleeding from the IVC by inserting a drainage cannula across the injured lesion. We think this procedure represents a novel treatment option for retrohepatic IVC injuries.

## Background

 The mortality associated with blunt liver injury has decreased by 0.2% per year to 7.3%,^1^ and more than 90% of these injuries can be treated with nonoperative management (NOM).^2^ However, concomitant retrohepatic inferior vena cava (IVC) injury is a fatal injury, with a mortality rate of 67%.^3 4^

The treatment of retrohepatic IVC injury requires laparotomy for hemostasis. Hepatic vascular exclusions and shunting to bypass the injury are generally poorly tolerated and rarely performed.^5 6^ Other options include gauze packing, direct repair of injured veins, and lobar resection.^7^ Gauze packing is the first choice in many cases of hemodynamic instability.^5^ However, laparotomy itself may attenuate the packing effect and promote bleeding. Additionally, excessive hepatic gauze packing reduces venous return to the right atrium, preload, and cardiac output due to venous pooling. Although stent grafts have been reported to be effective for IVC injuries,^8 9^ there is a risk of branch occlusion. A recent study has shown that the combination of balloon occlusion for the aorta and IVC contributed to the maintenance of circulatory dynamics.^10^ However, it is a bridge strategy until the injured site is repaired. Less invasive treatment of retrohepatic IVC injury without laparotomy and without inducing hemodynamic collapse due to reduced venous return may further improve outcomes in severe liver injury.

Recently, several reports have demonstrated the efficacy of extracorporeal membrane oxygenation (ECMO) for trauma patients.^11^ We incidentally encountered a case of an iatrogenic retrohepatic IVC injury that was successfully treated with the new concept of flow diversion effect using venoarterial ECMO. In this case, we hypothesized that negative drainage pressure on ECMO may have contributed to the successful treatment of the IVC injury. We also examined the impact of negative drainage pressure by ECMO on limiting hemorrhage after IVC injury in an animal model.

## Case presentation

The patient was a woman in her 80s with no specific background disease or past medical history. She suddenly lost consciousness at home, and her family called for emergency medical services. Shortly after the arrival of the ambulance, the patient experienced cardiopulmonary arrest (CPA). Endotracheal intubation and chest compression were performed during transportation to our hospital.

**Figure 1:**
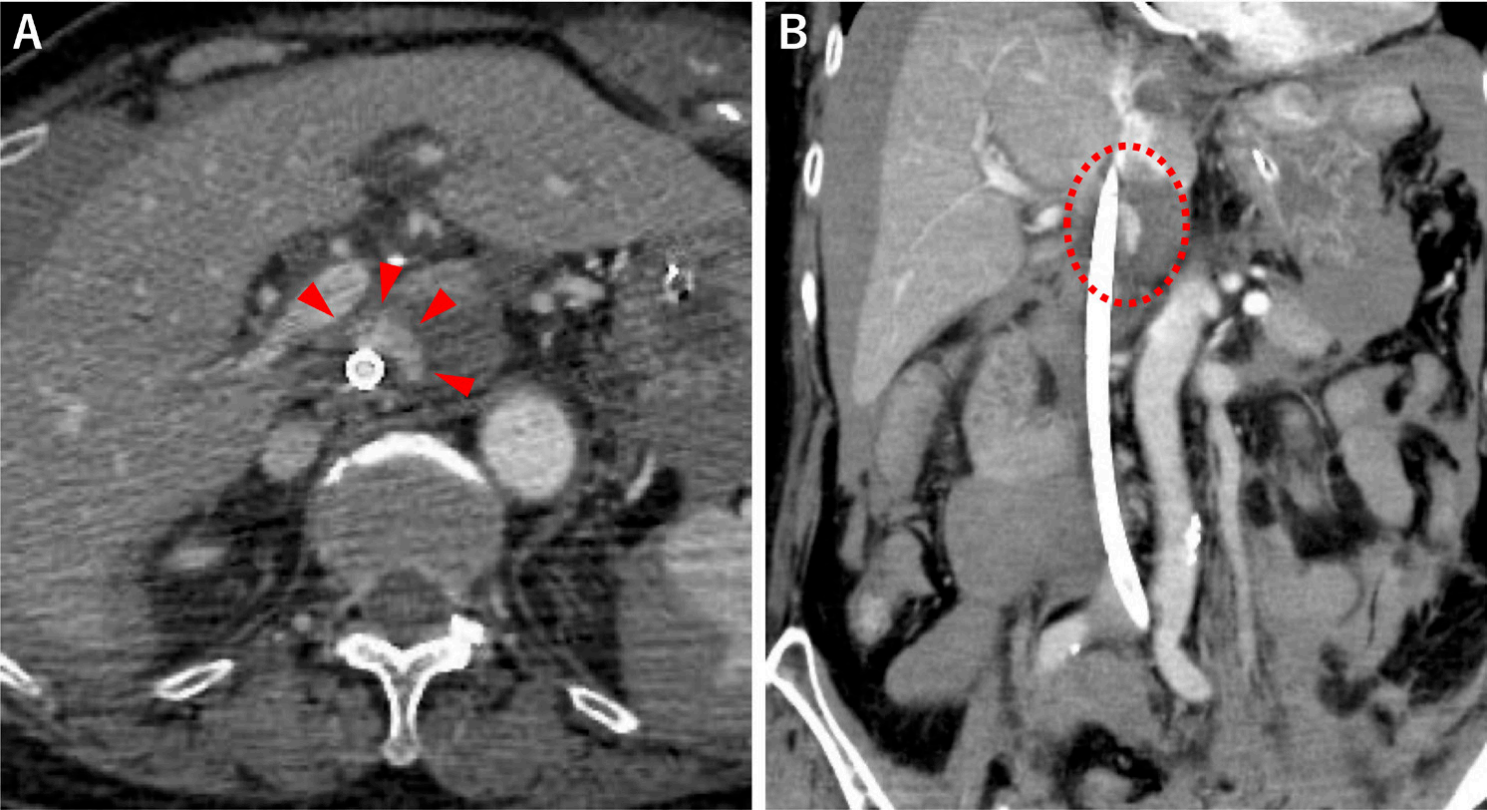
Contrast-enhanced CT after initiation of ECMO. (**A**) Axial view, (**B**) Coronal view. A 20-Fr drainage cannula for ECMO was inserted from the femoral vein to the right atrium. Contrast-enhanced CT showed extravasation of contrast medium around the IVC ((A) arrowhead, (B) dotted circle), suggesting retrohepatic IVC injury. The drainage cannula was located across the injured area. CT, computed tomography; ECMO, extracorporeal membrane oxygenation; IVC, inferior vena cava.

Spontaneous circulation was restored 4 minutes after CPA when the patient arrived at the hospital. A pulmonary artery embolism was suspected because of the D-shape on echocardiography. Chest–pelvis contrast-enhanced CT revealed massive thrombosis in both main pulmonary arteries. She underwent CPA again within 22 minutes of arriving at the hospital shortly after the CT. A 20-Fr drainage cannula and a 16-Fr return cannula (PCKC-VTM; MERA, Tokyo, Japan) of ECMO were inserted from the right femoral vein and the left femoral artery, respectively. Venoarterial ECMO was established 15 minutes after CPA. Subsequently, a transcatheter thrombectomy of the pulmonary artery was performed. Ultrasonography performed immediately after endovascular treatment showed the presence of ascites. Follow-up chest–pelvis contrast-enhanced CT after transcatheter thrombectomy revealed a hematoma on the chest wall and a laceration with extravasation of the contrast medium in the left lobe of the liver that was absent on initial CT, which was thought to be caused by the chest compressions. Additionally, a hematoma with extravasation of contrast medium was noticed around the IVC (figure 1). Left hepatic artery embolization was performed to treat the liver injury under ECMO. When the ECMO flow was increased to 3.5 L/min, blood immediately drained from the ECMO circuit, and IVC angiography confirmed there was no extravasation. Based on these findings, we concluded that IVC blood drainage by ECMO controlled the hemorrhage from IVC injury. We selected NOM for IVC injury. Anticoagulation therapy with heparin helped control the activated clotting time within 160 to 180 seconds. The ECMO drainage pressure was controlled by adjusting the rotational speed of the centrifugal pump to 2800 to 3200 rpm, which did not cause drainage failure and maintained a negative pressure.

As right ventricular dysfunction improved on echocardiography and the blood pressure was maintained even with reduced ECMO flow, ECMO was withdrawn on day 6. Subsequently, she developed a wound infection at the ECMO insertion site that required antimicrobial therapy. The patient was transferred to the hospital for rehabilitation on day 64.

### Verification by animal experiments

We subsequently verified our hypothesis that negative drainage pressure on ECMO can aid in the successful treatment of IVC injury in an animal model. We used a beagle dog (2 months old/male, 10.0 kg) to reproduce our findings. The dog was maintained in accordance with the guidelines of the Committee on Animal Studies at the National Cerebral and Cardiovascular Center, and the study was approved by the Animal Investigation Committee. The animal received humane care in compliance with the ‘Guide for the Care and Use of Laboratory Animals’ published by the National Institutes of Health.

**Figure 2:**
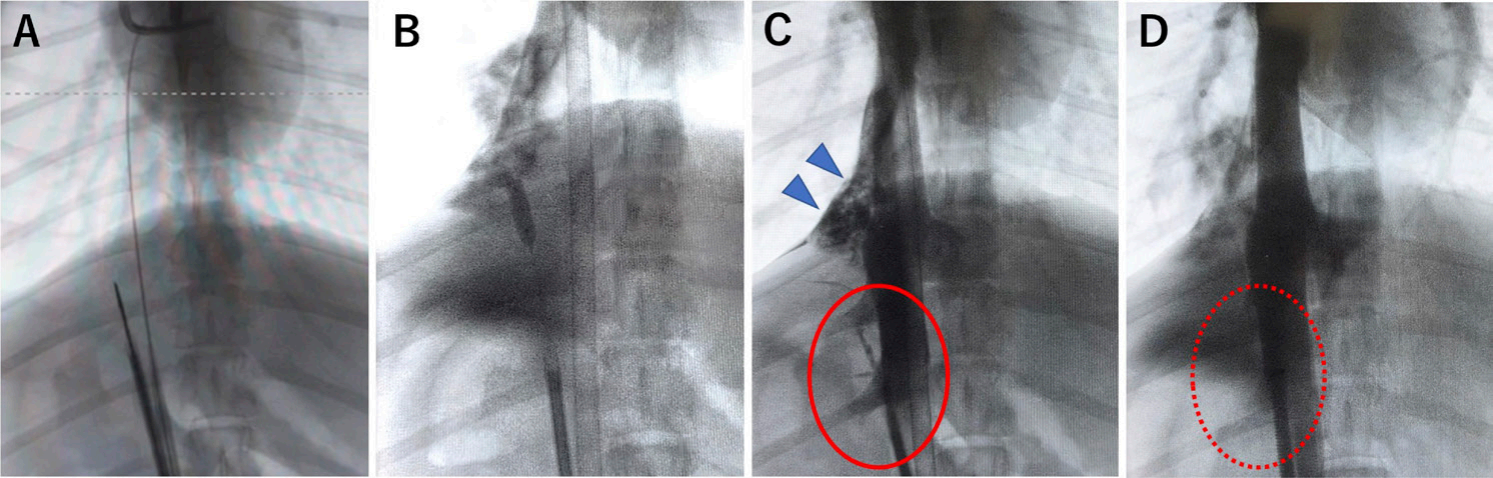
Animal model (beagle dog) of retrohepatic IVC injury. We used a beagle dog to reproduce and confirm the case findings. (**A**) The IVC was perforated using a Rösch-Uchida needle (Cook). (**B**) The perforation was dilated using a percutaneous transluminal angioplasty balloon. (**C**) IVC angiography showed extravasation of the contrast medium (circle) pooled under the diaphragm (arrowhead). (**D**) The IVC resolved after ECMO (dotted circle). ECMO, extracorporeal membrane oxygenation; IVC, inferior vena cava.

Under general anesthesia, a 12-Fr drainage cannula (CAPIOX; Terumo, Tokyo, Japan) was inserted from the left femoral vein into the right atrium and a 10-Fr return cannula was inserted from the left femoral artery as a standby for venoarterial ECMO. Retrohepatic IVC perforation was induced by inserting a Rösch-Uchida needle (Cook, Bloomington, USA) into the right femoral vein (figure 2A). The perforation was further dilated using a 3 mm percutaneous transluminal angioplasty balloon (ZINRAI; KANEKA Corp, Osaka, Japan) to simulate IVC injury (figure 2B). After inducing the injury, we confirmed massive extravasation of contrast medium around the region (figure 2C) and then initiated VA-ECMO. As the ECMO flow increased from 0 to 2.0 L/min, pressure at a drainage cannula increased gradually to −40 mm Hg. IVC angiography showed that the extravasation of contrast medium from the IVC injury had disappeared, consistent with the findings observed in our patient (figure 2D). The VA-ECMO flow and hemodynamic status were stable for 1 hour until the observation was terminated.

## Discussion

The findings from the patient and the animal model showed the possible application of ECMO to control active bleeding from IVC. In a previous report,^12^ utilizing ECMO for IVC injuries, a short stent graft was placed in the injured area, and venovenous ECMO was introduced to treat respiratory failure. However, the tip of the drainage cannula was placed near the renal vein, peripheral to the injured site.

In this case, we treated retrohepatic IVC injury using a completely different concept. Generally, venous blood flow is passive and does not depend on pulsation but on pressure gradients. Hence, bleeding can be controlled without repairing the injured area by facilitating blood flow to the side of the drainage cannula for insertion across the injured site. This concept was supported by the findings not only from our animal model but also from a previous report on a common iliac vein injury^13^ that showed a bare metal stent alone could control active bleeding. This effect is known as the ‘flow diversion effect’ and is used to treat cerebral aneurysms.^14^ The drainage negative pressure of ECMO may have had a direct positive effect on the injured area^15^ by decreasing venous pressure. Hence, this ECMO strategy, based on the flow diversion effect, allows for a less invasive treatment without the need for laparotomy or stenting. It can also be applied to the treatment of other venous injuries.

There may be concerns regarding the risk of increased bleeding due to trauma because anticoagulants are used during ECMO. However, a recent systematic review revealed comparable rates of bleeding and clotting with and without anticoagulants,^16^ suggesting that anticoagulants are not essential for ECMO. In this case, since the primary indication was pulmonary embolism, anticoagulation was continued during ECMO. Additionally, venovenous ECMO theoretically does not affect cardiac output. ECMO has been widely used to treat trauma patients.^11^

This study has some limitations. First, in the patient, a 20-Fr drainage cannula of approximately the same diameter as the IVC diameter calculated from CT was used, which had the risk of further widening the IVC. In the future, fluoroscopic cannulation should be considered. Second, several factors, such as blood volume, vessel diameter, and injury size, which may affect the outcomes, were not evaluated. Further detailed studies conducted under different conditions are required to validate our findings.

## Conclusions

ECMO for retrohepatic IVC injuries is a new conservative treatment method that can control bleeding through the flow diversion effect by inserting a drainage cannula to cross the injured area and adjusting the drainage negative pressure.
